# Synthetic Flavonoid 3,7-Dihydroxy-Isoflav-3-Ene (DHIF) Reduces In-Stent Restenosis in an Atherosclerotic Watanabe Heritable Hyperlipidemic Rabbit Stent Model

**DOI:** 10.3390/ijms252111530

**Published:** 2024-10-27

**Authors:** Jarkko P. Hytönen, Olli Leppänen, Jouni Taavitsainen, Seppo Ylä-Herttuala

**Affiliations:** 1 A.I. Virtanen Institute, University of Eastern Finland, 70210 Kuopio, Finland; jarkko.hytonen@uef.fi (J.P.H.);; 2 Heart Center, Kuopio University Hospital, 70200 Kuopio, Finland

**Keywords:** antioxidant: redox, superoxide, NF-kappaB, vessel injury, phytochemical, phytoestrogen, restenosis, rabbit model

## Abstract

Inflammation is a major component of the pathogenesis of atherosclerosis and the formation of in-stent restenosis (ISR). A novel flavonoid, DHIF, attenuates reactive oxygen species and nf-κB signaling and has potential to limit ISR via antioxidant action. While current drug eluting stents (DESs) perform well in clinical practice, new therapies to prevent ISR without dependance on cytotoxic drugs are warranted. Our objective was to test whether DHIF reduces ISR in a hyperlipidemic rabbit aorta model of ISR via attenuated inflammatory responses. WHHL rabbit aortas (*n* = 24) were denuded. Six weeks after injury, stents were implanted into the denuded aortas. DHIF was dissolved in carboxymethyl cellulose (CMC) and administered orally with two doses. CMC served as a control. The animals were sacrificed six weeks after stenting. ISR was evaluated from stent histomorphometry and immunohistology was used to assess the inflammatory and antiproliferative effects of the treatment. ISR was reduced from 20.9 ± 3.0% in controls to 15.2 ± 2.4% (*p* = 0.0009) and 16.4 ± 2.1% (*p* = 0.004) in the low- and high-dose groups, respectively. The neointimal area covered by macrophages was 32 ± 9.3% in the controls, 17.2 ± 5.9% (*p* = 0.005) in the low-dose group and 19.4 ± 7.9% (*p* = 0.008) in the high-dose group. DHIF significantly reduces ISR and local inflammation in stented arterial regions and could be used to reduce ISR when bare metal stents are used. Targeting local inflammation in the arterial wall may provide a way to reduce ISR in a clinical setting and further studies are warranted.

## 1. Introduction

Coronary artery stenting is a standard treatment for stabile and acute obstructive coronary artery disease [1,2]. The cytostatic drug eluting stents (DESs) reduce in-stent restenosis (ISR) through the inhibition of smooth muscle cell (SMC) proliferation and matrix secretion; nevertheless [3,4], earlier generations of DES have also been shown to prolong endothelial dysfunction, induce neoatherosclerosis and increase the risk of late stent thrombosis after coronary intervention [5,6]. Stent thrombosis is usually prevented with the use of dual antiplatelet therapy (DAPT) following treatment with a DES. Many patients are, however, at a high risk of bleeding while on DAPT and alternative therapeutic approaches are warranted. Although newer generations of DES have demonstrated improved safety and completely bioabsorbable devices have entered the market, it is vital to further investigate the mechanism of restenosis as well as exploring alternative solutions for the prevention and treatment of restenosis [5].

A controlled level of reactive oxygen species (ROS) and a healthy endothelium are essential for vascular homeostasis [6,7,8,9]. A percutaneous coronary intervention procedure with a stent causes injury to the endothelium and the deeper layers of the vessel wall, and this injury induces abundant ROS production [3,7,10]. An excess of ROS and the dysfunction of the endothelium contribute significantly to the progression of ISR. Both the restoration of antioxidant balance and the induction of re-endothelialization reduce ISR [8,11,12].

Flavonoids are a group of plant-derived chemicals, or phytochemicals. Due to their phenyl structure, flavonoids possess substantial antioxidant capacity in vitro. The ability of flavonoids to decrease oxidative stress in vivo, however, is suggested to result from the regulation of intracellular signaling and gene expression [13]. Isoflavones, a subgroup of flavonoids, resemble 17-estradiol in structure, and therefore may conduct estrogen agonist or antagonist action within the body, depending on concentration [14]. A novel synthetic flavonoid, 3,7-dihydroxy-isoflav-3-ene (DHIF), has been shown to attenuate NF-κB, prevent elevation in the ROS level, reduce proliferation and induce apoptosis in the neointima after arterial injury [15,16]. All these features are central in reducing ISR. Nevertheless, the efficacy of DHIF for the treatment of ISR has not been previously studied.

In this study, our objective was to examine the effects of oral treatment with DHIF on ISR in an atherosclerotic WHHL rabbit aorta after stent placement. The effects of DHIF administration are examined by determining the inflammatory response post stenting and the development of the neointima six weeks after stent deployment. We hypothesized that an attenuated inflammatory response would, in turn, be associated with reduced neointima formation.

## 2. Results

### 2.1. In-Stent Restenosis

**Figure 1:**
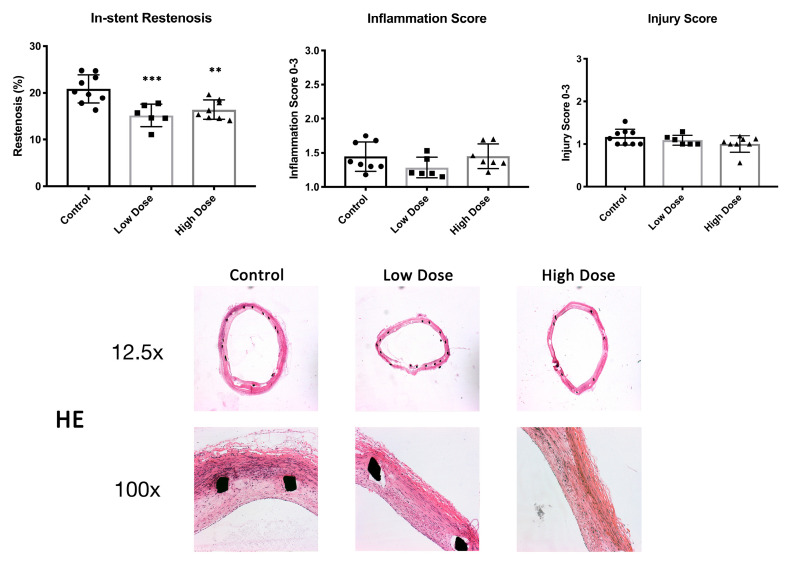
In-stent restenosis, inflammation and injury scores. In-stent restenosis was significantly reduced in the treatment groups compared to the controls (top left graph and HE histology). There were no differences in injury or inflammation scores at d42 determined from HE histology (top middle and right graphs). Data in graphs are shown as mean ± SD. Significance in graphs: ** *p* < 0.01; *** *p* < 0.001.

After the 42-day follow-up, histological restenosis in the stented artery was 20.9 ± 3.0% in the control animals. DHIF-treated animals showed a significantly lower ISR of 15.2 ± 2.4% and 16.4 ± 2.1% (low dose, *p* = 0.0009, and high dose, *p* = 0.004, respectively). There were no differences in the injury scores between the groups with scores of 1.2 ± 0.2, 1.1 ± 0.1 and 1.0 ± 0.2 (control, low dose and high dose, respectively; low dose *p* = 0.66; high dose *p* = 0.13) (Figure 1). Descriptive statistics and effect size estimates are shown in Table 1.

### 2.2. Inflammation, Proliferation and Apoptosis

**Figure 2:**
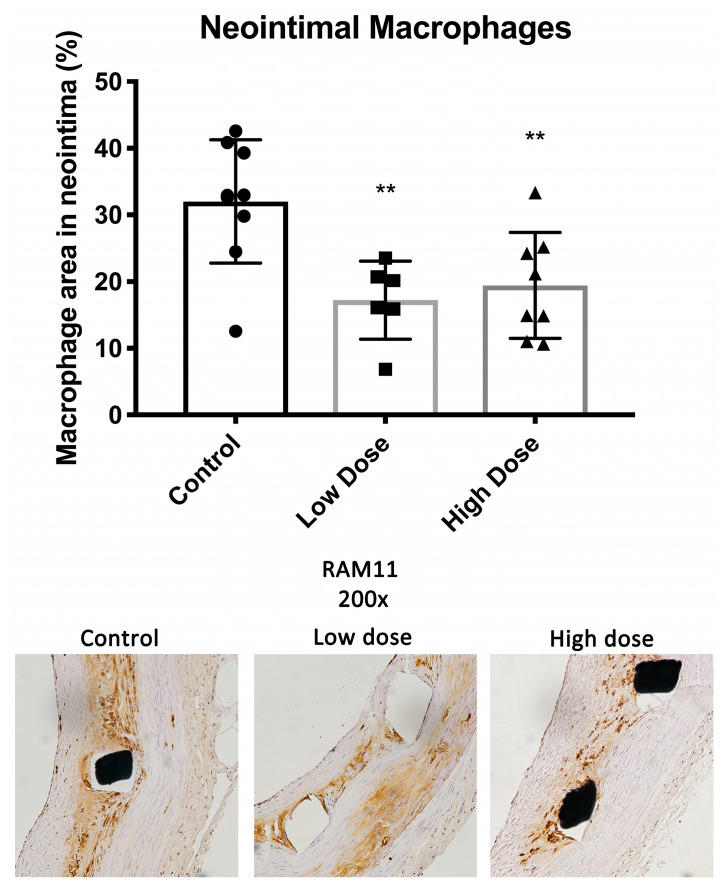
Neointimal macrophages. Macrophage areas measured in the neointimas of the treatment group stents (RAM-11 staining) were significantly smaller compared to the controls. Data are shown as mean ± SD, significance ** *p* < 0.01.

The neointimal area covered densely with macrophages was 32 ± 9.3% in the controls, 17.2 ± 5.9% (*p* = 0.005) in the low-dose group and 19.4 ± 7.9% (*p* = 0.008) in the high-dose group (Figure 2). There were no differences in the inflammation scores of 1.5 ± 0.2, 1.3 ± 0.2 and 1.5 ± 0.2 (control, low dose and high dose, respectively; low dose *p* = 0.22; high dose *p* = 0.99) (Figure 1).

**Figure 3:**
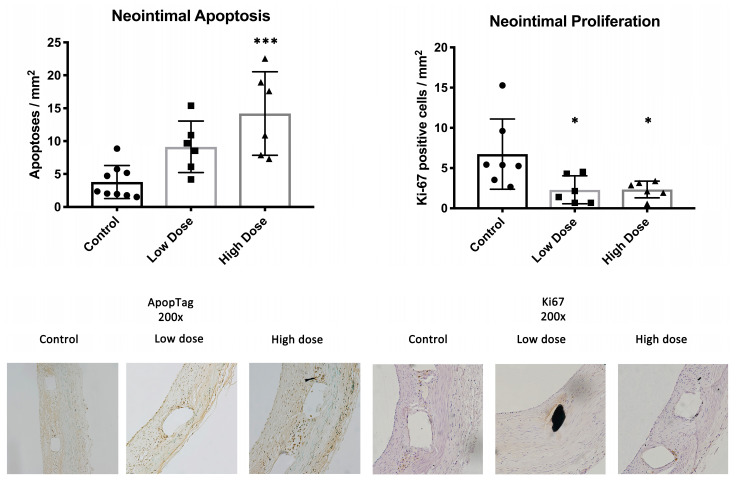
Apoptosis and proliferation. The high-dose treatment group showed increased apoptosis in the neointimal tissue at d42. Proliferation was attenuated in the neointimas of both treatment groups compared to the controls. Stainings from ApopTag kit for apoptosis and Ki-67 for proliferation. Data are shown as mean ± SD; significance: * *p* < 0.05; *** *p* < 0.001.

In the neointima, there were on average 7.2 ± 5.2, 2.3 ± 1.7 (*p* = 0.03) and 2.7 ± 1.1 (*p* = 0.03) proliferating cells per square millimeter in the control, low-dose and high-dose groups, respectively. The number of apoptotic cells in the control CMC-treated animals was 3.8 ± 2.5 per mm^2^ of neointima. For the treatment groups, the numbers of apoptoses were 9.1 ± 3.9 (*p* = 0.05) and 14.2 ± 6.3 (*p* = 0.0004) cells/mm^2^ (low dose and high dose, respectively) (Figure 3).

### 2.3. Endothelium

**Figure 4:**
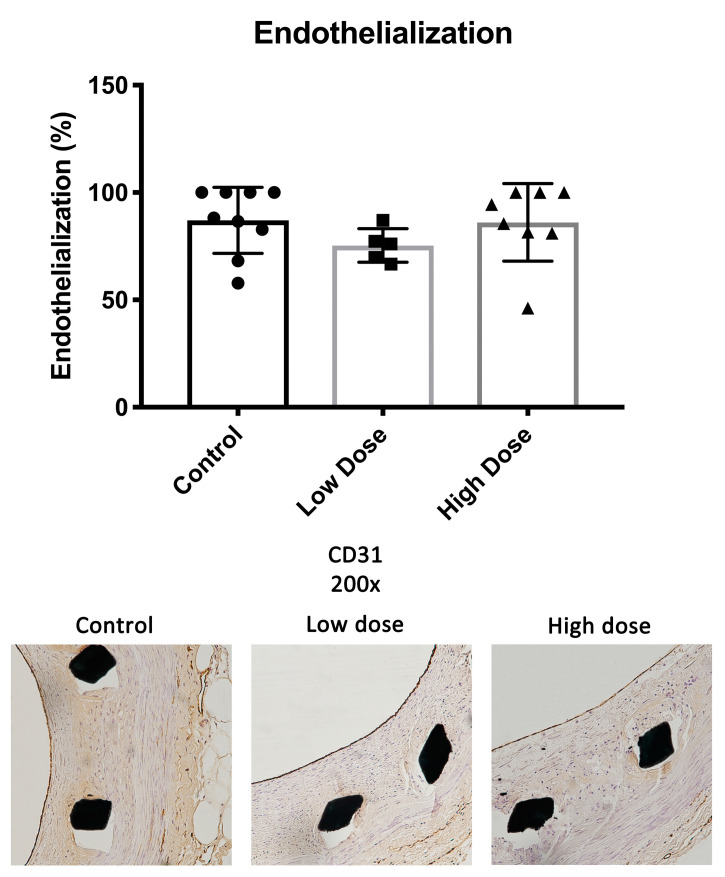
Endothelialization. No differences were seen in the rate of endothelial recovery by d42 between the study groups. Complete re-endothelialization was not observed in any of the groups. Data are shown as mean ± SD.

There were no significant differences in the endothelial coverage between the study groups. The endothelial coverage of the total lumen perimeter was 87.1 ± 15.4% in the controls, 75.4 ± 7.9% in the low-dose group and 86.1 ± 18.1% in the high-dose group (low dose *p* = 0.32; high dose *p* = 0.98) (Figure 4). 

### 2.4. Clinical Chemistry and Safety Tissue Histology

There were no significant differences between the groups in clinical chemistry analyses. Safety tissue histology presented no adverse effects related to the therapy (Table 2). 

### 2.5. Pharmacokinetics

**Figure 5:**
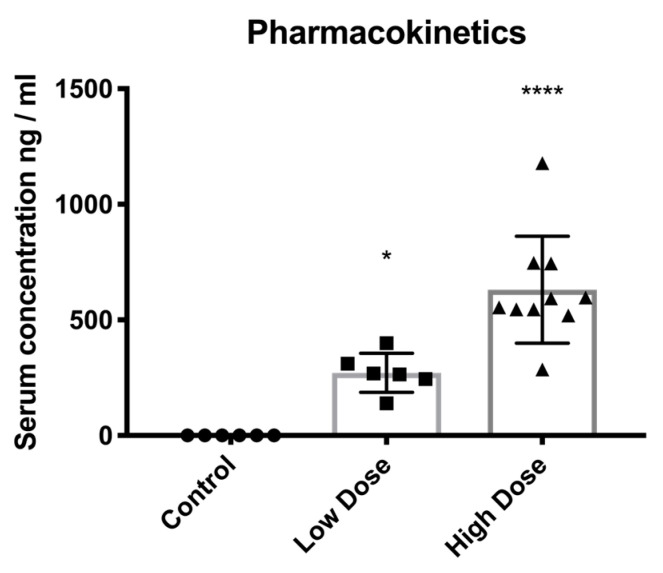
Pharmacokinetics. Levels of the study compound were measured one hour after the drug administration. Levels show a dose-dependent plasma concentration. Data are shown as mean ± SD; significance: * *p* < 0.05; **** *p* < 0.0001.

One hour after drug administration, the serum concentrations of DHIF were 271.1 ± 84.7 ng/mL (*p* = 0.018) and 630.8 ± 230.9 ng/mL (*p* < 0.0001) in the low- and high-dose groups, respectively. No drug was detected in the control animals (Figure 5). 

## 3. Discussion

In this study, a synthetic isoflavone, DHIF, significantly reduced ISR six weeks after stent deployment in a clinically relevant atherosclerotic WHHL rabbit model. In addition, treatment with DHIF in both dosage groups reduced the macrophage coverage of the neointima to approximately half of the coverage observed in the controls. This is highly important as inflammation is a key pathogenic factor in atherosclerosis and ISR [17,18,19]. Neointimal macrophages induce SMC proliferation and medial SMC migration into the neointima, both of which increase the volume of the neointima. The complicated atherosclerotic process is still centered on the properties and function of macrophages and smooth muscle cells when in contact with cholesterol [20,21,22,23]. This interplay of SMCs and macrophages in the presence of cholesterol led us to choose this highly relevant atherosclerotic model to conduct the present study in. Neointimal macrophages also promote the development of atherosclerotic vulnerable plaques, which are prone to rupture [3]. Furthermore, DHIF treatment induced apoptosis in the neointimal tissue while reducing proliferation.

Vascular injury causes ROS production to exceed compensatory antioxidant mechanisms and distorts the delicate ROS balance in the artery wall. This distortion results in inflammation and SMC proliferation [10]. Hence, redox regulation may explain the anti-inflammatory effects of DHIF observed in this study.

DHIF has been shown to attenuate NF-κB signaling in injured carotid artery. NF-κB family is a major regulator in injury, inflammation, cell proliferation and cell death [16,24]. All of these processes have a crucial role in the progression of ISR. Flavonoids’ ability to decrease oxidative stress in vivo is proposed to result from the regulation of intracellular signaling and gene expression [25]. There is evidence that isoflavones upregulate antioxidant gene expression through the modulation of NF-κB and Nrf2 [13]. An NF-κB decoy eluting stent reduced ISR in hypercholesterolemic rabbits [16].

The endothelial coverage was greater than 75% in our three study groups with no significant differences between the groups. The finding suggests that DHIF treatment causes no delay in endothelial healing six weeks after injury. This successful healing is important as healthy endothelium restricts leukocyte infiltration and SMC proliferation and thus arrests the processes of ISR and neoatherosclerosis. A healthy endothelium also protects from potential in-stent thrombosis. Overall, the extent of re-endothelialization observed in this study is comparable to what we have observed in previous experiments with the WHHL-ISR model [26]. 

There were no significant differences in ISR or macrophage infiltration of the neointima between the two daily doses of 25 mg/kg and 50 mg/kg of DHIF. In addition, both dosages were equally effective in reducing proliferation in the neointima. Thus, a daily dose higher than 25 mg/kg had very little impact on the treatment outcome. While we observed more apoptotic cells in the neointimas of the high-dose animals, this did not translate into better treatment outcomes with regard to ISR formation. 

A limitation of the study is the lack of a DES group as an additional control. The majority of stents implanted today have a drug coating and this will affect the microenvironment within the neointima. A DES study with a prolonged follow-up is warranted to study the effects of this therapy on neoatherosclerosis in DES.

## 4. Materials and Methods

### 4.1. Denudation and Stent Deployment

Adult Watanabe heritable hyperlipidemic (WHHL) rabbits (*n* = 24) weighing 3.1 to 3.6 kg were kept on a standard diet [27]. A denudation injury of the entire aorta was performed with a 3F Fogarty embolectomy catheter (Edwards Lifesciences, Irvine, CA, USA) introduced by femoral artery cut-down [26]. Six weeks after the injury, a bare metal stent (Trimax, Abbott Vascular, IL, USA) introduced by a carotid artery cut-down was deployed into an infrarenal abdominal aortic segment free of side branches, documented by angiography. The rabbits received 40 mg aspirin in drinking water starting two days before stenting. Clopidogrel was administered p.o., starting with a 30 mg loading dose on the day of stenting, followed by 15 mg daily. Enoxaparin was administered at a dose of 1 mg/kg s.c. daily from stenting [28]. Animals were sacrificed 42 days after stent deployment. Aortic stented segments and safety tissues from the heart, lung, liver, spleen, kidney and semimembranosus muscle as well as segments of the proximal and distal aorta in relation to the stent were collected and further processed for analysis. Instrumentations and euthanasia were performed under medetomidin (Domitor, 0.3 mg/kg, Orion, Espoo, Finland) and ketamine (Ketalar, 20 mg/kg, Pfizer, NY, USA) anesthesia. All animal experiments were authorized by the national Animal Experiment Board in Finland (ELLA).

### 4.2. Drug Administration

DHIF was purified by silica gel chromatography, followed by confirmation of the purity by mass spectrometry–liquid chromatography. DHIF was dissolved in 1% carboxymethyl cellulose (CMC) solution for administration. Animals were randomized into control group receiving CMC only, and two study groups receiving 25 mg/kg or 50 mg/kg (low dose and high dose, respectively) of DHIF daily. The daily dose was divided into two portions equal in size administered every twelve hours with an orogastric tube. Animals were weighed once a week for accurate dosing. The first dose was administered two days before stent implantation and administrations were continued until sacrifice. 

### 4.3. Blood Sampling

Arterial blood samples of six milliliters were collected from each rabbit before the first drug administration, before and after stent implantation and at sacrifice. Serum total cholesterol, LDL, HDL, creatinine, liver enzymes and high sensitivity-C Reactive Protein (hsCRP) were determined from the samples. Clinical chemistry analysis was performed at the Eastern Finland laboratory center. In addition, arterial blood samples were collected two weeks after stent implantation, one hour after drug administration, to evaluate DHIF drug serum levels. 

### 4.4. Tissue Processing

The stents were paraformaldehyde-fixed and embedded in methylmethacrylate plastic resin (Technovit^®^ 9100, Heraeus Kulzer GmbH, Division Technique, Wehrheim, Germany) [12]. Safety organ tissues were embedded into paraffin. Embedded stent and safety samples were prepared into 5–7 μm thick sections and stained. The histological stent sections represent the middle section of the stents.

### 4.5. Immunohistochemistry

Immunohistochemistry was performed with monoclonal antibodies (mAbs) against endothelium (CD31, 1:50, Agilent Dako, CA, USA), SMCs (HHF35, 1:50, Enzo, NY, USA), macrophages (RAM-11, 1:200, Dako) and proliferating cells (Ki-67, 1:100, Dako, with citrate buffer antigen retrieval). Apoptosis analyses were performed from sections with the ApopTag-kit (Millipore, MA, USA). Images were captured using an AX70 microscope (Olympus Optical, Japan) and further processed for publication with Photoshop (Adobe, CA, USA), adjusting brightness and contrast evenly over each image. 

### 4.6. Histomorphometry

The proportional ISR was determined as the ratio of neointimal area to the area of the internal elastic lamina {[1 − (Luminal area/IEL area) × 100]} from HE-stained sections of the cross-sections of the stented aortas at ×25 magnification. The proportion of intact endothelium of artery lumen perimeter was measured from CD31 immunostained sections of the stented aortas at ×40 magnification. The area covered with dense immunostaining with RAM11 per neointimal area was quantified from sections at ×200 magnification covering the entire intima. The number of proliferating cells per area was quantified from Ki67-immunostained histological sections at ×400 magnification. All histomorphometric analyses were performed using analysis software (Soft Imaging system, version 1) in a blinded manner. Pathological changes in safety tissues were assessed from HE-stained histological sections.

### 4.7. Statistical Analysis

All data represent mean ± standard deviation (SD). The statistical significance of the results is conveyed by values of *p* < 0.05 (*), *p* < 0.005 (**), *p* < 0.001 (***) and *p* < 0.0001 (****). Statistical significance was evaluated using GraphPad Prism software (version 10, GraphPad, MA, USA). Data normality was assessed with Shapiro–Wilk test and the data further analyzed with one-way ANOVA followed by Dunnett’s post hoc test. Cohen’s d values were determined for effect size analysis. 

## 5. Conclusions

In this study, a novel synthetic isoflavone, DHIF, significantly reduced ISR six weeks after stent deployment in a clinically relevant atherosclerotic WHHL rabbit model. The administration of DHIF significantly reduced inflammation and proliferation in the restenotic lesions and did not delay endothelial recovery after the stenting injury. In the days of modern DESs, new concerns, especially regarding the need for dual antiplatelet therapy for high-bleeding-risk patients, have arisen. Therefore, new therapies without cytotoxic agents that impair endothelial recovery are in demand. Safety and the ease of oral administration make DHIF a potent drug candidate to treat restenosis.

## Figures and Tables

**Table 1:** Descriptive statistics and effect size estimates.

**In-Stent Restenosis**				**Neointimal Apoptosis**			
	Control	Low Dose	High Dose		Control	Low Dose	High Dose
Mean	20.9	15.2	16.4	Mean	3.8	9.1	14.2
Median	20.3	15.2	15.8	Median	2.4	9.1	14.2
Range	8.5	6.7	5.5	Range	7.4	11.2	15.2
Cohen’s d		1.6	1.3	Cohen’s d		1.4	2.0
**Endothelialization**				**Pharmacokinetics**			
	Control	Low Dose	High Dose		Control	Low Dose	High Dose
Mean	87.1	75.4	86.1	Mean	0.0	271.1	630.8
Median	88.2	76.0	90.0	Median	0.0	266.5	573.7
Range	42.2	20.4	53.8	Range	0.0	259.1	891.5
Cohen’s d		0.7	0.1	Cohen’s d		4.5	3.9
**Neointimal Macrophages**				**Injury Score**			
	Control	Low Dose	High Dose		Control	Low Dose	High Dose
Mean	32.0	17.2	19.4	Mean	1.2	1.1	1.0
Median	32.9	18.1	18.1	Median	1.1	1.1	1.0
Range	30.0	16.7	22.7	Range	0.5	0.3	0.6
Cohen’s d		1.5	1.2	Cohen’s d		0.3	0.7
**Neointimal Proliferation**				**Inflammation Score**			
	Control	Low Dose	High Dose		Control	Low Dose	High Dose
Mean	6.7	2.3	2.3	Mean	1.4	1.3	1.5
Median	5.4	1.8	2.5	Median	1.4	1.2	1.4
Range	12.6	3.8	2.8	Range	0.6	0.4	0.5
Cohen’s d		0.9	0.8	Cohen’s d		0.7	0.0

Summary of descriptive statistics for the evaluated parameters and effect size evaluation with Cohen’s d values.

**Table 2:** Clinical chemistry.

	Control (CMC)		Low-Dose DHIF		High-Dose DHIF	
	d0	d42	d0	d42	d0	d42
**ALT**	22.3 ± 10.0	23.0 ± 5.9	31.3 ± 10.5	23.2 ± 3.9	26.6 ± 19.5	26.9 ± 18.5
**ALP**	41.3 ± 20.7	37.8 ± 13.1	34.0 ± 7.5	28.5 ± 5.5	36.8 ± 11.7	32.3 ± 8.7
**AST**	15.3 ± 4.5	17.3 ± 7.3	27.0 ± 13.7	18.3 ± 3.6	21.0 ± 9.8	22.9 ± 9.8
**GT**	2.5 ± 2.1	2.7 ± 0.9	5.2 ± 0.7	5.6 ± 1.1	7.6 ± 7.1	6.6 ± 3.6
**Bilirubin**	0.7 ± 0.3	0.5 ± 0.3	0.4 ± 0.2	0.6 ± 0.3	0.4 ± 0.4	0.8 ± 0.3
**Creatinine**	59.0 ± 11.3	53.7 ± 5.8	66.7 ± 17.5	64.2 ± 17.9	83.6 ± 15.3	87.6 ± 25.1
**Cholesterol**	15.1 ± 4.9	15.8 ± 6.0	12.1 ± 9.7	12.9 ± 9.5	17.3 ± 2.6	16.1 ± 4.7
**Triglycerides**	8.6 ± 8.4	9.8 ± 6.7	6.6 ± 0.7	5.3 ± 1.7	9.8 ± 3.5	7.4 ± 4.7
**hS-CRP**	0.12 ± 0.03	0.12 ± 0.02	0.10 ± 0.02	0.12 ± 0.02	0.11 ± 0.01	0.10 ± 0.02

No significant changes were observed in standard laboratory tests between the study groups or between d0 and d42. Serum levels of ALT (Alanine Aminotransferase), ALP (Alkaline Phosphatase), AST (Aspartate Aminotransferase), GT (Gamma-glutamyltransferase), bilirubin, creatinine, total cholesterol, triglycerides and High Sensitivity C-Reactive Protein (hS-CRP) were determined.

## Data Availability

The original contributions presented in the study are included in the article, and further inquiries can be directed to the corresponding author.
